# Critical coupling in plasmonic chain for efficient energy trapping

**DOI:** 10.1038/s41598-025-05446-7

**Published:** 2025-07-01

**Authors:** Marius Crouzier, Fei Mao, Giovanni Magno, Vy Yam, Carlos Alonso-Ramos, Jean-René Coudevylle, Etienne Herth, Christophe Dupuis, Xavier Leroux, Thomas Lopez, Béatrice Dagens

**Affiliations:** 1 Université Paris-Saclay, CNRS, Centre de Nanosciences et de Nanotechnologies, Palaiseau, 91120 France; 2 Department of Electrical and Information Engineering, Polytechnic University of Bari, Via Orabona, 4, Bari, 70125 Italy; 3 Centre technique de Vélizy, Stellantis, Vélizy-Villacoublay, 78140 France

**Keywords:** Plasmons, Integrated waveguide, Critical coupling, Temporal coupled-mode theory, Nanoparticle chain, Nanophotonics and plasmonics, Sub-wavelength optics, Integrated optics, Nanoparticles

## Abstract

Plasmonic nanoparticles can concentrate energy at the nanometer scale, offering promising applications across multiple fields such as lab-on-chip technologies and photonic circuits. A crucial requirement for these applications is achieving efficient coupling between the nanoparticles and the excitation signal. Plasmonic nanoparticle chains can guide light at subwavelength scale and can be excited through coupling to a dielectric waveguide. In this manuscript, we propose a novel configuration for the plasmonic chain-dielectric waveguide structure that allows the chain to be freely positioned relative to the waveguide. We demonstrate the existence of a critical coupling regime between a silicon waveguide and a plasmonic chain, achieved through precise control of their separation. In this regime, the plasmonic chain transitions from its well-known transmission mode to a new cavity state, trapping 99% of the waveguide’s energy. This result paves the way for efficiently addressing nanostructures through integrated waveguides, enabling efficient optical nano-tweezers, sensors or nano-heaters.

**Supplementary Information:**

The online version contains supplementary material available at 10.1038/s41598-025-05446-7.

## Introduction

Trapping energy at subwavelength scale is essential for the development of compact integrated devices. Localized surface plasmons (LSP) have the ability to concentrate energy in subwavelength spots in metallic nanoparticles^1,2^. Especially, plasmonic chains, consisting of a series of metallic nanostructures, are particularly powerful yet simple structures. They enable the implementation of numerous miniaturized functions such as nano-heaters^3,4^ nanotweezers^5–7^ nanoantennas^8–10^ in photonic circuits. Indeed, plasmonic chains occupy a unique position in the plasmonics landscape. They exhibit a dual behavior, combining optical guiding capabilities with resonant dipole properties^11,12^. The chain supports both near and far-field interaction between nanoparticles, enabling energy propagation along its length. This allows the waveguiding of light by resonant dipole coupling, without requiring additional confinement layers^13,14^. Such a waveguide experiences high optical losses, leading to a broad operational bandwidth of the composing resonators, which is further intensified by their coupling along the chain^15,16^.

Furthermore, the coupling between a resonator and its excitation source can be tuned to achieve critical coupling^17^, a specific regime where the resonator absorption rate equals its energy input rate. This leads to a complete energy transfer from the excitation signal to the resonator mode, without any residual transmission or reflection at the system ports. This property has been used not only to achieve highly efficient coupling with nanoantennas^18,19^ but also to reach near-perfect absorption^20^ (optical switch^21,22^ amplitude/phase modulator^23^). In addition, critical coupling also leads to an abrupt transmittance phase shift between the resonator response and its excitation, at the resonance frequency. This phase shift is very sensitive to the surrounding environment making this phenomenon very promising for sensing application^24,25^.

A plasmonic chain integrated near a dielectric waveguide, whatever the shape of the plasmonic nanostructures, the geometry of the waveguide and the light polarization state, can be considered as a system consisting of two coupled waveguides, enabling efficient individual excitation of plasmonic nanostructures by waveguide coupling mechanism^11,26,27^. It can also be considered as a system made of a dielectric waveguide coupled to a collectively resonant structure. From this point of view, a critical coupling regime is also expected in this system.

In this context, we have studied the possibility of achieving critical coupling between a plasmonic chain and a dielectric waveguide TE mode. Using experimental, theoretical and numerical approaches, we demonstrate that the chain exhibits different collective excitation regimes. The predominance of one regime over the other can be continuously tuned by adjusting the system geometry. We show that the critical coupling enables complete confinement of waveguide mode energy within the chain, resulting in a sharper spectral response that can be controlled by engineering design parameters.

The manuscript is organized as follows: (i) we first describe the investigated device and its characteristics. Then, (ii) we present the key experimental results obtained from optical transmittance measurement through the functionalized waveguide. (iii) These experimental findings are then compared with FDTD simulations. Subsequently, (iv) we apply the temporal coupled-mode theory (TCMT) framework to shed light on the underlying physics of the system. Finally, (v) we discuss these results in detail, providing physical insights through dispersion curve analysis.

## Results

### Experimental results

**Fig. 1:**
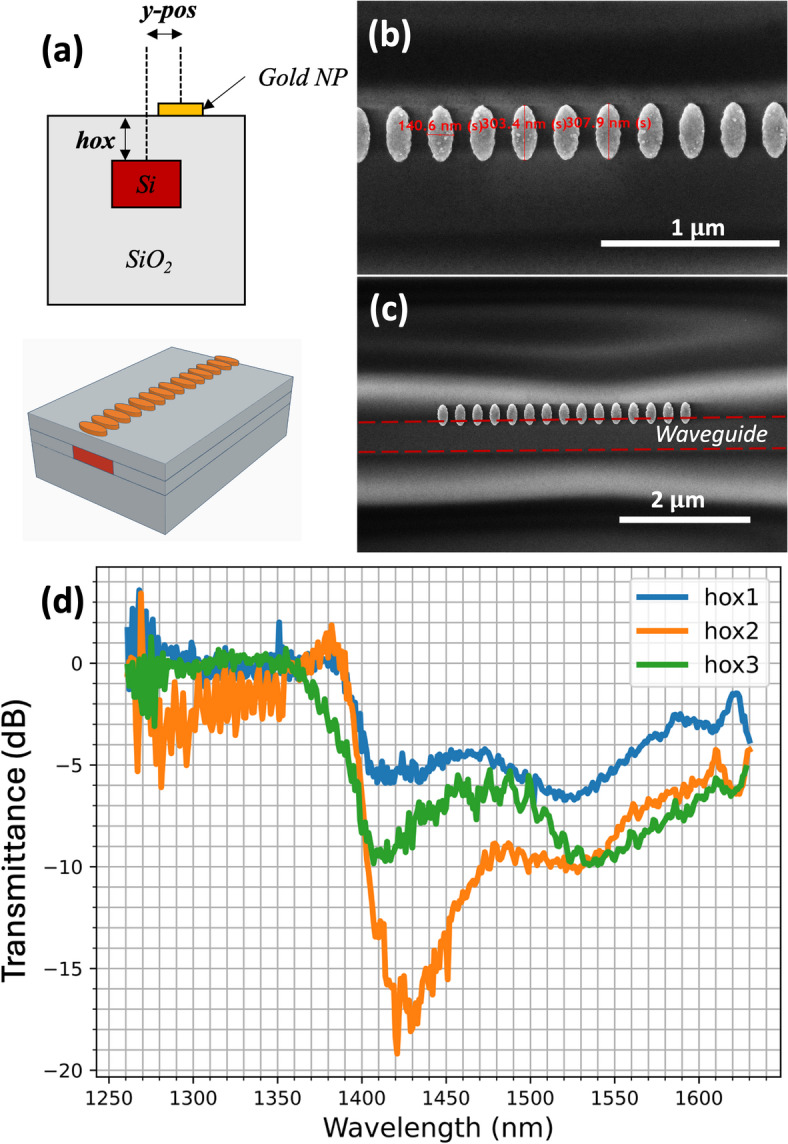
(**a**-**b**) Sketch of the structure investigated. The Si waveguide is encapsulated in a SiO_2_ cladding. The cladding height hox can be adjusted during the fabrication process. The resulting flat surface above the waveguides enables nanoparticle chains to be positioned with a lateral shift *y-pos* relative to the waveguides. (**c**-**d**) SEM images of the H1 sample, with *y-pos* = 410 nm. (**d**) Measured waveguide transmittance for 15 NP chains and different values of *hox*: *hox1* = 35 $\:\pm\:$ 5 nm, *hox3* = 75$\:\pm\:$ 5 nm, and *hox2* is between these two values. The minimum transmittance is reached at *hox* = *hox2*. The lateral shift is constant for the three chains *y-pos*$\:\:=\:$300 nm (sample H1).

The investigated structure combines a Silicon-on-Insulator (SOI) waveguide with a periodic chain of gold nanostructures aligned parallel to the waveguide direction (Fig. 1a). The waveguide height is 220 nm, and its width is 450 nm. It is encapsulated in a silica layer, allowing the free positioning of the metallic nanostructures near the waveguide. Using standard FDTD simulations, we designed the device to ensure the plasmonic resonance of the chains operates within the range of 1260–1630 nm. The silica thickness between the top of the waveguide and the SiO_2_ surface is named *hox*. As illustrated in Fig. 1b-c, each nanoparticle has an ellipsoidal shape with a height of 30 nm and a periodicity of 238 nm. The chains comprise either 10 or 15 nanoparticles, with their central axis offset from the SOI waveguide axis by distance *y-pos*. The nanoparticles long axis is perpendicular to the waveguide axis, allowing the excitation of the nanoparticle LSP by the evanescent field of the TE mode in the waveguide. The fabrication process (S1) allows precise control of the chain-to-waveguide distance through *hox* and *y-pos* parameters, enabling modulation of coupling strength between the TE mode and the chain propagating plasmonic mode.

We fabricated two distinct samples containing several functionalized waveguides. The sample labeled H1 features varying *hox* heights, while the sample labeled H2 has a constant *hox*. The waveguide encapsulation process involves HSQ resist deposited by spin coating, followed by annealing and etching (S2). Due to spin coating boundary effect, the resist thickness exhibits a non-uniform planarity, creating variations that persist after etching. This results in a slightly increased height at the sample center compared to its corners. For H1, we exploited this height variation by distributing waveguides across the entire sample surface to achieve different *hox* values. In contrast, for H2, we placed the waveguides at the center to obtain a constant *hox* value.

We investigate the influence of the chain-waveguide distance on transmittance spectra. As it is well known, the plasmonic resonance of the chain manifests as a characteristic dip in the transmittance spectrum at the resonance wavelength^11^. Thus, we are investigating the coupling strength between the TE mode and the plasmonic mode through transmittance measurements.

Transmittance curves of sample H1 were measured using the setup described in S3. The waveguides contain 15-nanoparticle chains with a semi-major axis of 155 ± 1 nm and a semi-minor axis of 83 ± 1 nm. All waveguides have identical *y-pos* = 300 nm but different *hox* height. While these waveguides have the same abscissa, their ordinates are separated by 690 $\:{\upmu\:}m$ on the sample, resulting in different *hox* (i.e.: $\:hox1<hox2<hox3$) and consequently different coupling strengths. Exact *hox* values could not be measured without dicing the sample. However, ellipsometric measurements estimate the smallest and the largest heights to be *hox1 =* 35 ± 5 nm and *hox3* = 75 ± 5 nm, respectively. Figure 1d displays the transmittance spectra of the three waveguides. The transmittance dip is shallowest for the lowest *hox (= hox1)*. As *hox* increases (= *hox2*), the transmittance minimum initially deepens before becoming shallower again at the highest *hox* (= *hox3*). For *hox2*, the transmittance at the resonance reaches − 19.6 dB, exhibiting a sharper dip. Compared to a waveguide without a nanoparticle chain, 99% of the energy within the waveguide is absorbed at the resonance wavelength.

In sample H2, the transmittance was measured for structures with a constant height *hox*$\:=\:92\:\pm\:\:7$ nm but a lateral position of the chain central axis ranging from 0 nm to 350 nm. Each chain consists of 10 gold nanoparticles, with a semi-major axis of 165 nm and a semi-minor axis of 75 nm. The waveguide’s transmittance spectra show that the resonance has a maximum strength and width when the chain is centered on the waveguide. When the chain is laterally displaced, the transmittance minimum intensity first increases, then decreases to a second minimum at *y-pos =* 200 nm. Further lateral displacement causes the minimum to increase again.

**Fig. 2:**
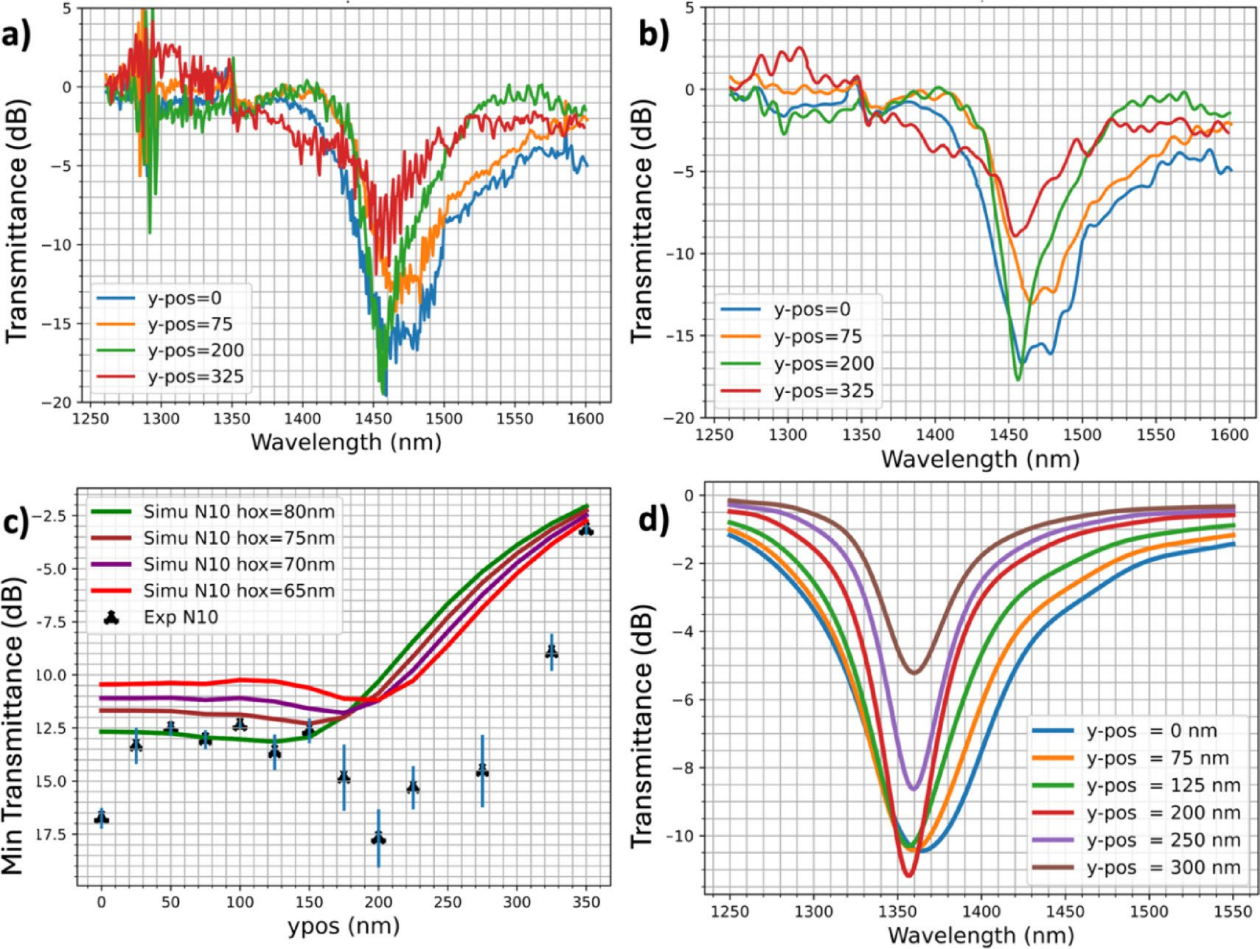
(**a**) Measured transmittance at different lateral positions (*y-pos*) of the 10 NP chain (sample H2). (**b**) Measured transmittance with high frequencies filtered for clarity. A 4^th^ order Butterworth digital filter has been used with a frequency cutoff ten times smaller than the sampling frequency. The filter has been implemented in Python in the library SCIPY^28^. (**c**) Minimum transmittance reached at the resonance for different values of *y-pos*: experimental values are represented by trefles and error bars corresponding to two times the transmittance standard deviation calculated. Curves are the minimum transmittance from FDTD simulations for different values of hox. (**d**) Transmittance calculated by FDTD simulation for *hox* = 65 nm and various values of *y-pos*, the resonance bandwidth reduces as the chain-waveguide distance increases.

In Fig. 2b, the transmittance spectra have been smoothed to improve their readability. As the chain is moved laterally away, the resonance bandwidth appears to continuously narrow. At *y-pos* = 200 nm, the resonance bandwidth is narrower than at *y-pos =* 0 nm, with comparable absorption.

The transmittance minima obtained in Fig. 2a are given in Fig. 2c as a function of *y-pos*. As the signal is varying near the wavelength resonance, these minima are displayed with error bars representing twice the transmittance standard deviation calculated in the wavelength window of 1445–1510 nm from the experimental raw data. These curves show constant values from *y-pos =* 25 nm up to *y-pos* = 150 nm, then a minimum at *y-pos* = 200 nm, and finally a strong increase at *y-pos*$\:\ge\:$ 275 nm.

### FDTD results

**Fig. 3:**
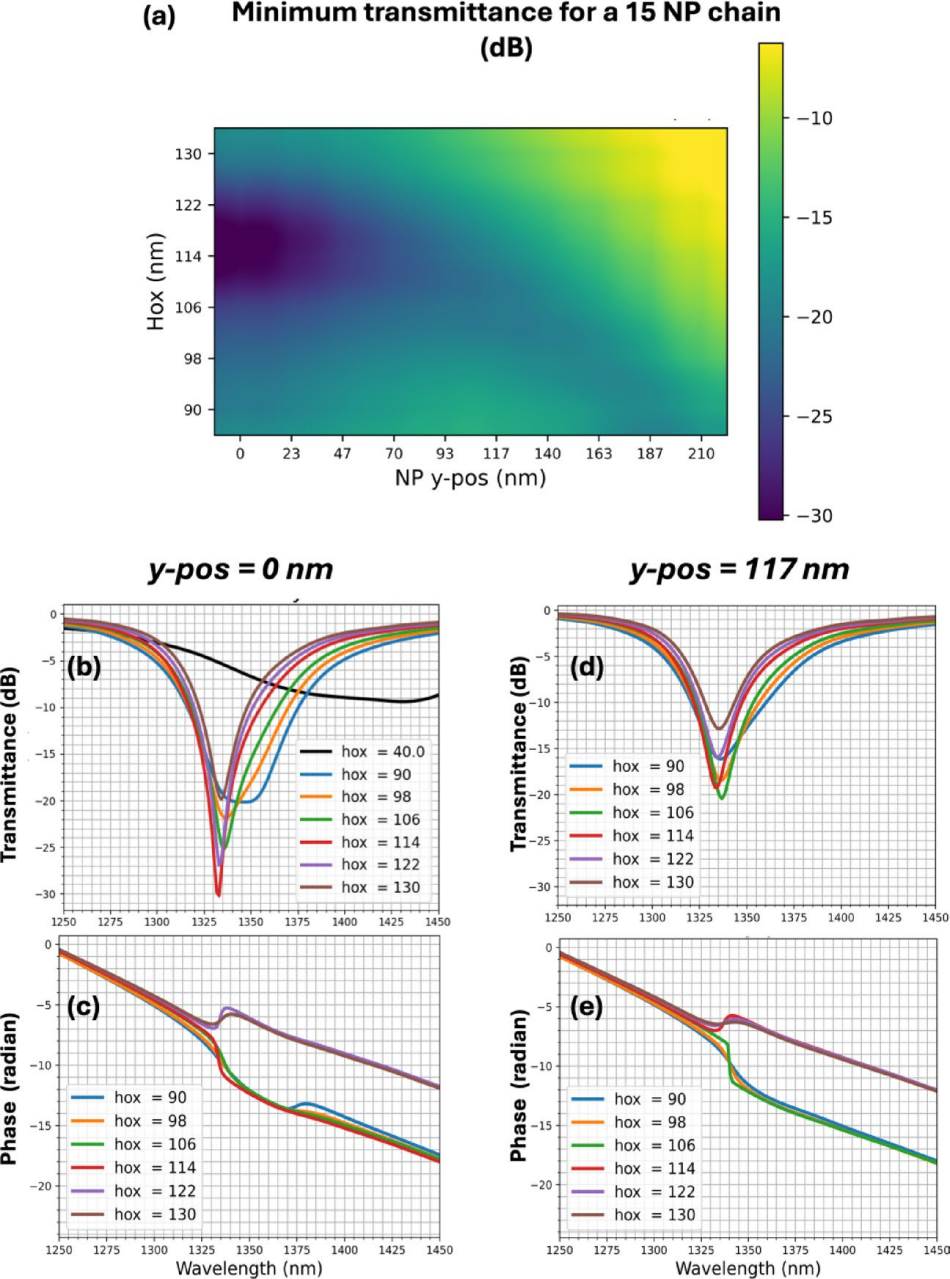
Simulation results for a 15-nanoparticle chain. Each nanoparticle has a semi-major axis of 145 nm, a semi-minor axis of 75 nm, with the chain having a 210 nm period. The SOI waveguide measures 220 nm in height and 450 nm in width. For the gold nanoparticles, dispersion data are determined by fitting a Drude model to ellipsometric measurements. Simulations employ a 3D mesh with minimum step of 3 nm in the chain region and in the silica between the waveguide and the chain. (**a**) Minimum transmittance obtained for different positions of the chain. (**b**-**c**) Transmittance and phase of the transmittance signal for different value of hox when the chain is centered on the waveguide and when it is laterally shifted (**d**-**e**).

To analyze these experimental results, we first performed FDTD simulations using a 10-nanoparticle chain with *hox* ranging from 65 nm to 80 nm. In Fig. 2c, the experimental results differ slightly from the simulations. While our measurements show a minimum at *y-pos* = 200 nm with *hox* $\:\approx\:$ 92 nm, simulations suggest that this minimum should occur at *hox* ≈ 65 nm. Higher *hox* values in the simulations result in minimum transmittance obtained for closer lateral positions (*y-pos*). In Fig. 3d, where the transmittance spectra for *hox* = 65 nm are plotted, the resonance wavelength exhibits a 100 nm blue shift compared to the experimental results. However, the minimum transmittance value is also reached for a lateral position *y-pos* around 200 nm. These discrepancies arise from variations in parameter values influencing the resonance between simulations and experimental results. Indeed, the nanoparticle height has a strong influence on the resonance wavelength (S4) and could differ between experiments and simulations for several reasons: nanofabrication deviations leading to height variations, surface roughness, and/or the non-perfectly flat surface where the nanoparticles are deposited. In addition, in simulations, gold is modeled by a Drude model where the parameters have been extracted from experimental measurement on continuous thin-film and not nanostructured gold in individual nanoparticles. Therefore, the resonance wavelength shift may result from a combination of these factors.

Furthermore, the resonance bandwidth reduces with increasing the chain-waveguide distance, which is consistent with the experimental results.

More detailed FDTD simulations were performed to gain a deeper understanding of the transmittance minimum evolution as the chain-waveguide distance is varied by *hox* and *y-pos*. A chain of 15 nanoparticles has been studied to emphasize the effect observed in Fig. 2.

Figure 3a maps the transmittance minima for various hox and y-pos values. The lowest transmittance minimum (−30 dB) occurs when the chain is centered on the waveguide (*y-pos* = 0) and at a specific height (*hox* = 114 nm, as confirmed in Fig. 3b). For hox below this value, shallower minima appear as the chain is moved laterally. For instance, at *y-pos* = 117 nm, a minimum of −20.5 dB is obtained at *hox* = 106 nm (see Fig. 3c). Notably, changes in *hox* or *y-pos* strongly modify the shape of the transmittance curve, which become deeper and sharper around these specific values.

Simulations also reveal a phase shift at resonance (see Fig. 3d, e).

The sign of this phase shift depends on whether the chain-waveguide distance is smaller or larger than the specific distance at which minimum transmittance occurs. The sign change is abrupt and highly sensitive to distance. These behaviors are characteristic of critical coupling phenomena^21,22,24^.

In our system, the chain-waveguide distance affects the coupling between the TE-guided mode and the plasmonic mode. This distance modification appears to alter the balance between coupling and losses, inducing a critical coupling phenomenon, despite the system comprises two coupled waveguides.

### Temporal coupled mode theory

**Fig. 4:**
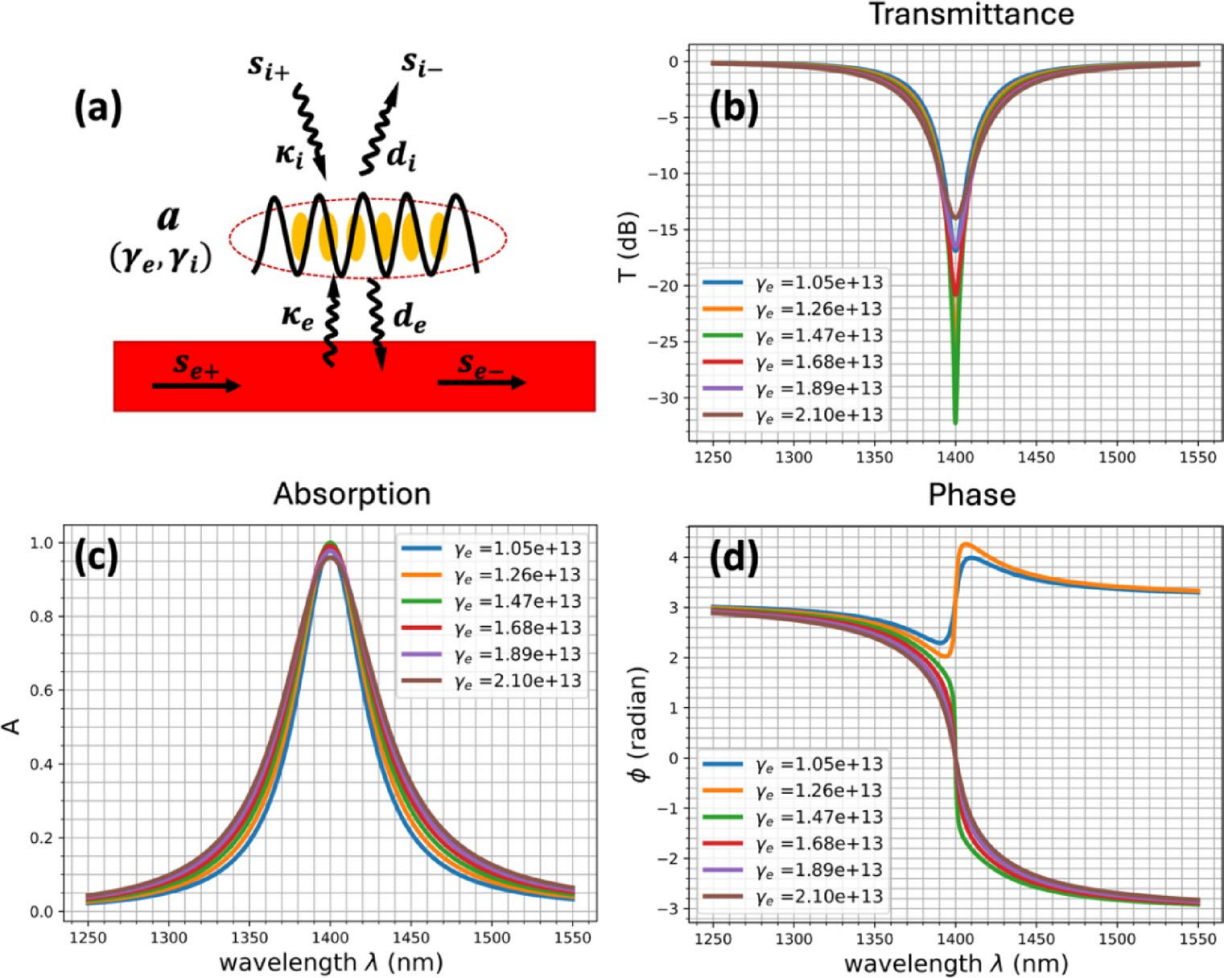
(**a**) Sketch of the model. The chain is a resonator exchanging energy through two different channels modeling the external coupling with the waveguide and the internal decay due to intern loss. (**b**) Transmittance $\:{\left|\frac{{\text{s}}_{\text{e}-}}{{\text{s}}_{\text{e}+}}\right|}^{2}$in decibels, (**c**) the absorbance $\:{\left|\frac{{\text{s}}_{\text{i}-}}{{\text{s}}_{\text{e}+}}\right|}^{2}$ and (**d**) the transmittance phase $\:{\upvarphi\:}$ calculated by the TCMT when the external decay rate $\:{{\upgamma\:}}_{\text{e}}$ is varying. The internal decay rate is constant: $\:{{\upgamma\:}}_{\text{i}}$= 1.4e13 s^-1^.

To obtain more insight on the behavior of the integrated chain we apply the TCMT to this system. It is a phenomenological approach broadly used in photonics to describe resonant systems and the energy exchanges between the different components^29,30^. In our system, the resonator is the nanoparticle chain exchanging energy with the waveguide. The chain has two energy exchange channels: $\:{s}_{i\pm\:}$ for the energy absorption and $\:{s}_{e\pm\:}$ for the energy exchanged with the waveguide (Fig. 4a). The resonator loss is described through the decay rate $\:\gamma\:={\gamma\:}_{i}+{\gamma\:}_{e}$ which is the sum of the intrinsic decay $\:{\gamma\:}_{i}$ and the extrinsic decay $\:{\gamma\:}_{e}$.

Since we represent the energy absorption as a channel input or output, we can consider that our system is energy conservative and has time reversal symmetry. This links the different decays to the coupling coefficients $\:{\kappa\:}_{i,e}$ and $\:{d}_{i,e}$to the decay rates through the following relations demonstrated in Supplementary Information^31^ (S6).

1.1 $$\:\begin{array}{c}{d}_{e}={{\kappa\:}}_{e}=j\sqrt{2{\gamma\:}_{e}}\end{array}$$

1.2 $$\:\begin{array}{c}{d}_{i}={\kappa\:}_{i}=j\sqrt{2{\gamma\:}_{i}}\end{array}$$

These relations are crucial for understanding the system’s behavior as it links the coupling strength between the waveguide and the chain to the external decay of the chain. Then, we can derive an expression for the chain’s absorbance A and the waveguide’s transmittance T at the resonance $\:({\upomega\:}={{\upomega\:}}_{0}$):

2 $$\:\begin{array}{c}A={\left|\frac{2\sqrt{{{\gamma\:}_{e}\gamma\:}_{i}}}{{\gamma\:}_{i}+{\gamma\:}_{e}}\right|}^{2}\end{array}$$

3 $$\:\begin{array}{c}T={\left|\frac{{{\gamma\:}_{i}-\gamma\:}_{e}}{{\gamma\:}_{i}+{\gamma\:}_{e}}\right|}^{2}\end{array}$$

When the external and internal decays are equal ($\:{\gamma\:}_{e}={\gamma\:}_{i}$), the system is in the critical coupling configuration: the absorbance reaches its maximum ($\:A=1$) and the transmittance is $\:T=0$.

The phase of the transmittance can be expressed as:

4 $$\:\begin{array}{c}\varphi\:=Arctan\left(\frac{2{\gamma\:}_{e}\left({\upomega\:}-{{\upomega\:}}_{0}\right)}{{\gamma\:}_{i}^{2}-{\gamma\:}_{e}^{2}+{\left({\upomega\:}-{{\upomega\:}}_{0}\right)}^{2}}\right)\end{array}$$

Near the resonance $\:{\upomega\:}\approx\:{{\upomega\:}}_{0}$, the phase has a singularity when the critical coupling condition is fulfilled ($\:{\gamma\:}_{e}={\gamma\:}_{i}$). Therefore, at the resonance, the phase defined as a function of $\:{\gamma\:}_{e}$ ($\:{\gamma\:}_{i}$ constant) is discontinuous for $\:{\gamma\:}_{e}={\gamma\:}_{i}$.

We calculated the transmittance, the absorption and the transmittance phase through the TCMT equations for different values of $\:{\gamma\:}_{e}$ (Fig. 4b-d). When $\:{\gamma\:}_{e}={\gamma\:}_{i}$, the transmittance reaches its lowest value, the absorption attains its maximum, and the transmittance phase-shift undergoes an abrupt sign change.

Furthermore, the resonance bandwidth $\:{\Delta\:}\omega\:$ is proportional to the decay rate $\:\gamma\:$, consequently, a decrease of $\:{\gamma\:}_{e}$ reduces the resonance bandwidth.

Therefore, these theoretical curves show the same behavior as the transmittance curves calculated in the FDTD simulation when the chain-waveguide distance is varying.

The distance *hox* between the waveguide and the NP chain significantly affects the coupling strength between the guided mode and the chain mode. The TCMT shows (Fig. 4) that the coupling strength is directly related to the chain’s extrinsic decay $\:{\gamma\:}_{e}$ and affects the transmittance minimum, the transmittance phase and the transmittance bandwidth in the same way as *hox* (Fig. 3). Indeed, $\:{\gamma\:}_{e}$ decreases as the NP chain moves away from the area near the waveguide.

This can also be observed through the numerically calculated dispersion diagrams of the system. Three different diagrams were calculated for varying values of *hox* in the case of an infinite gold nanoparticles chain. These band diagrams exhibit an anticrossing between the TE mode of the waveguide and the plasmonic mode of the chain. Their coupling forms two supermodes. As the height *hox* increases, the anti-crossing of the two modes diminishes, indicating that the coupling strength (related to $\:{\gamma\:}_{e})$ is weaker, thus demonstrating the influence of *hox* on $\:{\gamma\:}_{e}$.

**Fig. 5:**
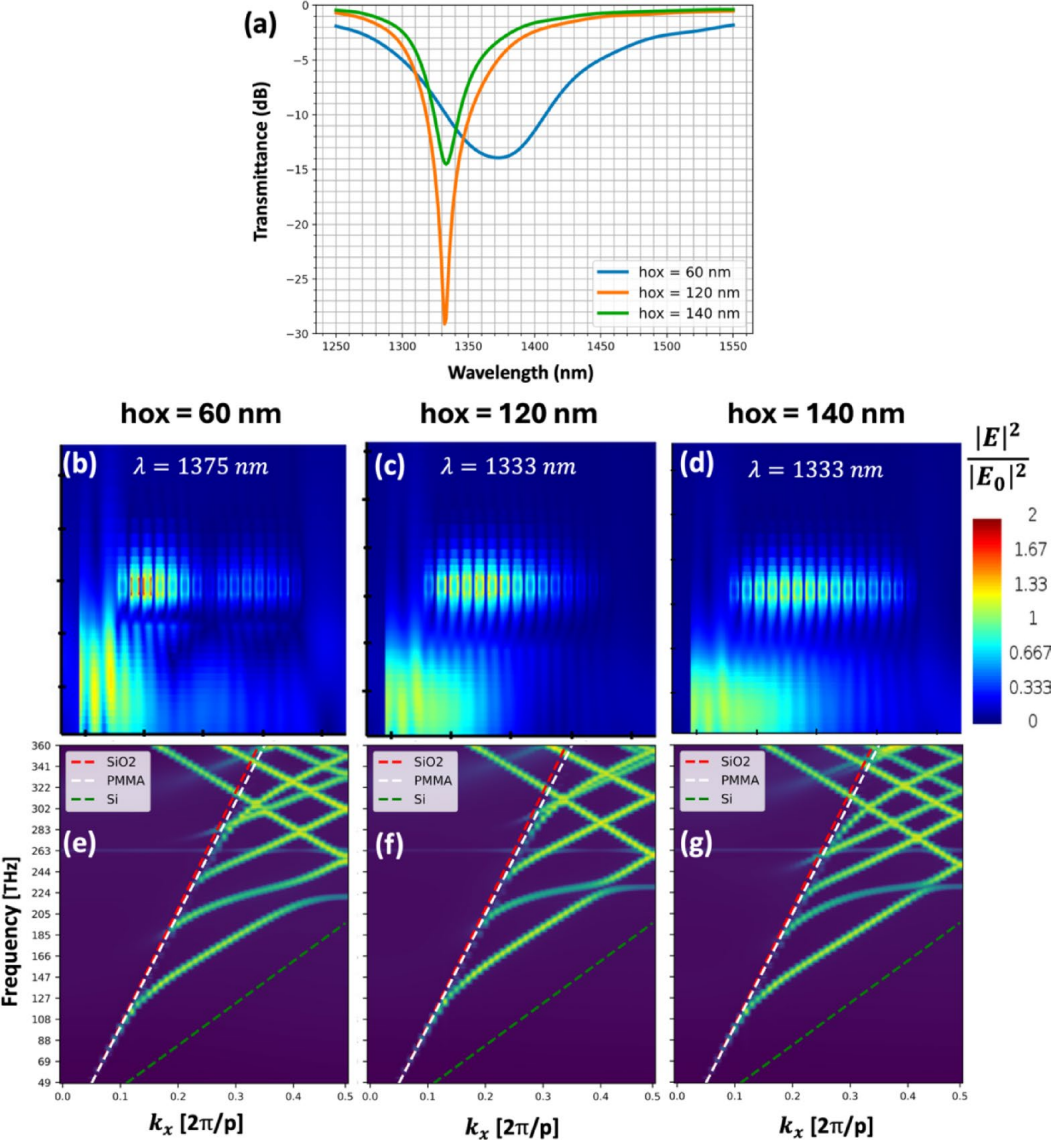
(**a**) FDTD calculation for a 15-nanoparticule chain for three different *hox*. (**b**-**d**) Electric field intensity distribution at the resonance wavelength for *hox* = 140 nm (**b**), *hox* = 120 nm (**c**), and *hox* = 60 nm (**d**). For *hox* = 60 nm, the energy oscillates between the chain and the waveguide. For *hox* superior at 120 nm, the coupled energy in the chain decreases exponentially along the chain. (**e**-**g**) Band structure of an encapsulated waveguide with an infinite gold nanoparticle chain centered on the waveguide (*y-pos* = 0) for different heights: (**e**) *hox* = 60 nm, (**f**) *hox* = 120 nm and (**g**) *hox* = 140 nm. The increase of the height reduces the anticrossing between the TE mode of the waveguide and the plasmonic mode of the chain and flattens the mode band.

Furthermore, the curves for both modes become progressively flatter near the anticrossing as the height increases. In a band structure, the curve slope corresponds to the group index of the mode which is inversely proportional to its group velocity in the medium. This flattening of the curve thus describes a strong decrease in the group velocity revealing that the mode no longer propagates in the chain.

The electric fields intensity distribution of the chain at the wavelength resonance for different values of *hox* (Fig. 5) also exhibits different excitation regimes. When *hox* = 60 nm, the transmittance curve of the structure shows a dip at the wavelength $\:{\uplambda\:}=\:1375\:nm$. The electric field intensity distribution shows that the energy is periodically exchanged between the waveguide and the plasmonic chain. The chain acts as a waveguide, and energy propagates through it as two beating supermodes.

At *hox* = 120 nm, the dip in the transmittance curves is deeper. The electric field intensity distribution shows that almost all the waveguide energy enters and is absorbed by the chain, and the energy no longer propagates within the system, which is consistent with the flattening of the dispersion curve. At *hox* = 140 nm, the intensity in the chain is concentrated on the first nanoparticles and decreases exponentially along the chain. However, the nanoparticles are less excited resulting in a shallower transmittance dip at the wavelength resonance compared to *hox* = 120 nm.

Consequently, by controlling the chain-waveguide distance, it is possible to engineer the coupling rates and thereby control the collective excitation regime of the chain. When the chain is close to the waveguide, the system is overcoupled ($\:{{\upgamma\:}}_{e}>{{\upgamma\:}}_{i}$), meaning that the energy coming from the waveguide mode exceeds the loss within the metallic chain. Therefore, the energy can be coupled back into the waveguide, and the plasmonic chain behaves like a waveguide. Increasing the chain-waveguide distance weakens the coupling strength $\:{\gamma\:}_{e}$, leading to a decrease in group velocity. As it approaches to zero, energy no longer propagates along the chain. In this regime, the nanoparticle chain effectively behaves like a localized resonator.

At a specific distance, the critical coupling condition ($\:{\gamma\:}_{e}={\gamma\:}_{i}$) is achieved. In this configuration, the balance between external and internal decay results in near-perfect absorption of the waveguide mode by the nanoparticle chain.

If the chain-waveguide distance is increased beyond the critical regime, the system transitions to the undercoupled regime ($\:{\gamma\:}_{e}<{\gamma\:}_{i}$). In this regime, while all the energy coupled into the chain is absorbed, the coupling strength $\:{\gamma\:}_{e}$is insufficient to extract all the energy from the waveguide. This results in an increased waveguide transmittance.

## Discussion

This analysis provides a framework for understanding the discrepancies between the experimental and simulated results observed in Fig. 3c. The critical coupling condition is highly sensitive to the variation of the different parameters involved. Firstly, the gold optical index in the simulations was obtained from ellipsometry measurements performed on a full-plate sample, not directly on gold nanoparticles. Discrepancies between the actual optical properties of the fabricated nanoparticles and the bulk values can significantly alter the value of both external $\:{\gamma\:}_{e}$ and internal $\:{\gamma\:}_{i}$ decay rates, leading to a discrepancy between the experimental and simulation results. Secondly, in contrast to simulations, the fabricated nanoparticles have a surface roughness, which increases the internal losses $\:{\gamma\:}_{i}.$ Thirdly, the surface of the encapsulation layer is not as flat as in the simulations. For all these reasons, the critical coupling condition is altered and occurs at different chain positions in the experiment compared to the numerical simulations.

## Conclusion

To conclude, we proposed a novel design with planar encapsulated waveguides enabling flexible 3D positioning of the plasmonic chain with respect to the waveguide. This configuration enables the precise control of the coupling between the waveguide’s TE mode and the chain’s plasmonic mode. Numerical and experimental demonstrations show the dual behavior of the nanoparticle chain: it can behave either as a waveguide or as a resonator depending on the coupling strength. In the critical coupling region, almost the entire waveguide mode energy is trapped in the chain, leading to sharp spectral responses, reduced group velocity and strong spatial confinement. This capability unlocks the potential for efficient integrated addressing of dense arrays of plasmonic nanostructures into planar systems, with promising applications in lab-on-chip technologies, such as nano-heaters, nano-tweezers. In addition, the slowdown of the group velocity increases light-matter interactions and could benefit catalytic amplification and sensors. Finally, coupling these structures with phase change materials could enable the development of advanced nano-tunable optical devices.

## Methods

### Sample fabrication

The manufacturing process (see Supplementary Information S1) starts with an SOI sample with a 220 nm thick silicon layer. The first step is to use electron lithography to pattern the waveguides design by using a ZEP resin mask. A Raith EPBG 5200 with an accelerating voltage of 100 kV, a dose of 280 µC/cm2, a current of 0.8 nA and a beam step size of 2 nm. The sample is then etched by ICP-DRIE using C_4_F_6_ and SF_6_ gas. Once the sample has been cleaned of resin residue, a 700 nm thick layer of HSQ was deposited by spin-coating. This resin is transformed into SiO_2_ by annealing in a tubular furnace under nitrogen atmosphere. At the beginning, the temperature is set at 200 °C, then it is steadily increased at a rate of 5 °C per minute until reaching 600 °C, where it stays for 1 h. The furnace is then cooled down over 2 h to reach a temperature around 400 °C. To obtain the desired height between the guides and the surface, the sample was etched with ICP-RIE sentech using CHF_3_ gas. Using a laser tracking method, the etched height is monitored in real time. Finally, a second electron lithography is performed to form the mask of the gold nanoparticles. This lithography is also performed with an EPBG 5200, with an accelerating voltage of 100 kV, a dose of 380 µC/cm2, a current of 0.8 nA, and a beam step size of 2 nm. The resist used for this lithography is a CSAR 200. A 3 nm thick titanium layer was first deposited via evaporation at 0.2 nm/s to ensure the correct adhesion of the gold. Then, gold deposition was also achieved via an evaporation at 0.1 nm/s step followed by a lift-off in butanone.

### Optical characterization

The light from a single-mode fiber is injected into the waveguide via a grating coupler. Transmittance is defined by the ratio of optical output power to the input power at the SOI waveguide ports, in decibel. The input power is delivered by a tunable laser with 1 nm step scanning in the range of 1260–1630 nm. A manual fiber polarization controller ensures the TE polarization. The grating coupler creates an envelope in the transmittance spectrum making difficult the observation of the resonance. To overcome this issue, the transmittance of the waveguides with a nanoparticles chain was normalized against that of a reference waveguide without plasmonic structures. In each sample, straight waveguides are gathered in groups of 6 (Supp Info S3). One of these waveguides, does not contain gold nanoparticles chain so that its transmittance spectrum exhibits only the grating envelope. The other waveguides have a transmittance spectrum exhibiting the grating envelope and the effect of the chain resonance. Their transmittance spectrum normalizing by the transmittance spectrum of the waveguide without nanoparticles allows the suppression of the grating envelope, and thus makes the chain resonance observable. In addition, during the encapsulation step of the fabrication process, the cladding height can vary over the sample surface. This may have impact on the grating performance by coupling less light or by changing the diffraction order wavelength. For this reason, each group of 6 waveguides includes a waveguide without nanoparticle, so that each waveguide is normalized by a waveguide with the same grating coupling.

## Electronic supplementary material

Below is the link to the electronic supplementary material.

Supplementary Material 1

## Data Availability

The datasets used and/or analysed during the current study available from the corresponding author on reasonable request.
