# Supplementary material for: Critical coupling in plasmonic chain for efficient energy trapping
Source: Sci Rep. 2025 Jul 1;15:22072. doi: 10.1038/s41598-025-05446-7 (PMC12214624; doi:10.1038/s41598-025-05446-7)
Supplement: Supplementary file 1 — Supplementary Material 1 [file 41598_2025_5446_MOESM1_ESM.pdf]

SUPPLEMENTARY INFORMATION

Critical Coupling in Plasmonic Chain for Efficient Energy Trapping

Marius Crouzier[1,3], Fei Mao[1], Giovanni Magno[2], Vy Yam[1], Carlos Alonso-Ramos[1], Jean-René Coudevylle[1], Etienne Herth[1], Christophe Dupuis[1], Xavier Leroux[1*], Thomas Lopez[3], Béatrice Dagens[1]

[1]*Université Paris-Saclay, CNRS, Centre de Nanosciences et de Nanotechnologies, 91120, Palaiseau, France*
[2]*Department of Electrical and Information Engineering, Polytechnic University of Bari, Via Orabona, 4, 70125, Bari, Italy*
[3] *Stellantis, Centre technique de Vélizy, 78140 Vélizy-Villacoublay, France*

CONTENT

S1: Sample fabrication

S2: Encapsulation characterization

S3: Methods for optical characterization

S4: Resonance wavelength dependance on nanoparticle height

S5: Waveguide's reflectance

S6: Derivation of the Temporal Coupled Mode Theory (TCMT)

References

S1: Sample fabrication

The manufacturing process (Fig. S1) starts with an SOI sample with a 220 nm thick silicon layer. The first step is to use electron lithography to pattern the waveguides design by using a ZEP resin mask. A Raith EPBG 5200 with an accelerating voltage of 100kV, a dose of 280 µC/cm2, a current of 0.8 nA and a beam step size of 2 nm. The sample is then etched by ICP-DRIE using C_4F_6 and SF_6 gas. Once the sample has been cleaned of resin residue, a 700 nm thick layer of HSQ was deposited by spin-coating. This resin is transformed into SiO_2 by annealing in a tubular furnace under nitrogen atmosphere. At the beginning, the temperature is set at 200°C, then it is steadily increased at a rate of 5°C per minute until reaching 600°C, where it stays for 1 hour. The furnace is then cooled down over 2 hours to reach a temperature around 400°C. To obtain the desired height between the guides and the surface, the sample was etched with ICP-RIE sentech using CHF_3 gas. Using a laser tracking method, the etched height is monitored in real time. Finally, a second electron lithography is performed to form the mask of the gold nanoparticles. This lithography is also performed with an EPBG 5200, with an accelerating voltage of 100kV, a dose of 380 µC/cm2, a current of 0.8 nA, and a beam step size of 2 nm. The resist used for this lithography is a CSAR 200. A 3 nm thick titanium layer was first deposited via evaporation at 0.2 nm/s to ensure the correct adhesion of the gold. Then, gold deposition was also achieved via an evaporation at 0.1 nm/s step followed by a lift-off in butanone.

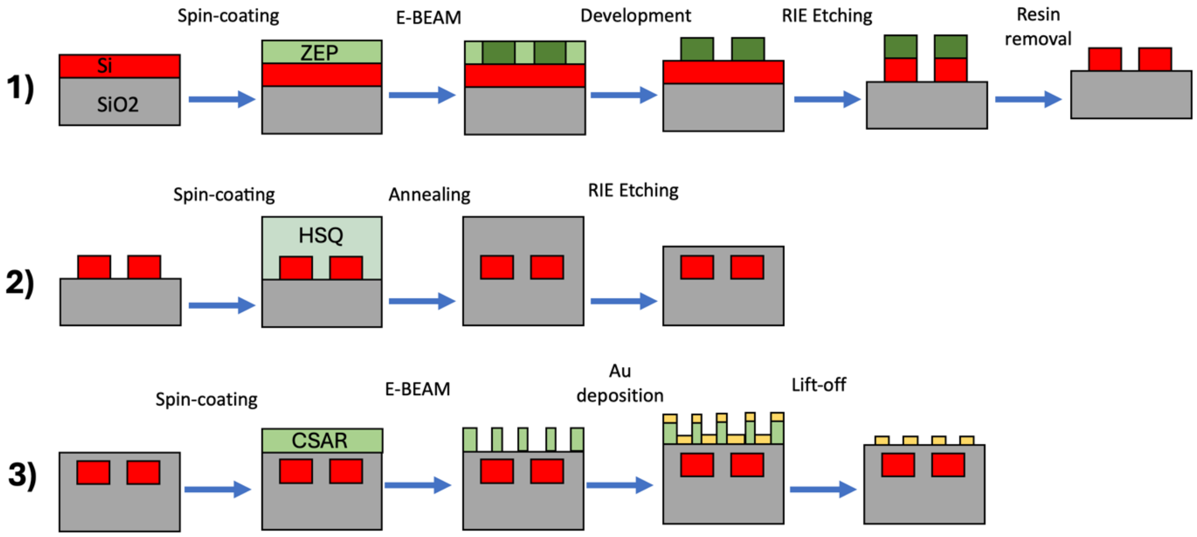

Figure S1: Fabrication process was composed of 3 main steps. The first consists in fabricating the Si waveguides by electronic lithography followed by an etching process. The second one was the encapsulation with a 700 nm layer of HSQ that is transformed in SiO_2 by annealing. The desired height above the waveguide is attained by etching this layer. The last step is for the gold nanoparticles fabrication, a mask is made by electronic lithography and the gold deposited by vapor evaporation step and a lift-off is performed.

S2 : Encapsulation characterization

The edge effects occurring during the spin-coating lead to non-uniform height on the entire sample surface (1cm x 1.2cm) [1,2]. All waveguides were in a 8.5 mm side square area. Each side of the waveguide's area is surrounded by 3 square marks of 1 mm² in size equally spaced by 2.5 mm along the waveguide's area side. One of these marks is at the center of the waveguide's area. These marks are used as reference marks for the ellipsometry measurements.

In Figure S2, the sample height was measured by ellipsometry at the different marks. The height is maximum in the center and minimum in the sample's corner. The surface exhibits a slope that is assumed to be constant. These measures give a first estimation of the height *hox*. However, the surface is not perfectly flat in the waveguides neighboring, so to obtain a more accurate estimation of *hox* above the waveguides, profilometry measurements are performed. Thus, the value of *hox* is estimated by combining the value from ellipsometry and profilometry measurements.

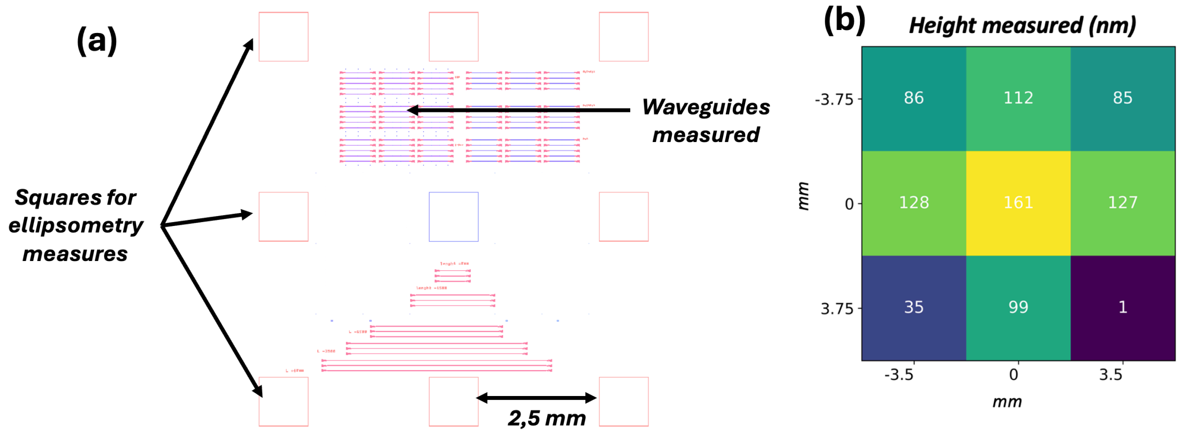

Figure S2: (a) gds file of the sample H1, all the structures are comprised in 8.5 mm by 8.5 mm area. The waveguides are surrounded by 1 mm² squares, and one is at the center of the structure. These squares define marks where to measure the height of the cladding in the same area at different steps of the fabrication process. (b) Heights measured in these squares after the encapsulation step. The height is more important at the center than at the border because of the boundary effects during the spin-coating process.

S3 : Methods for optical characterization

In Figure S3.1, the optical set-up used to measure the waveguide transmittance is illustrated. The light from a single-mode fiber is injected into the waveguide via a grating coupler. Transmittance is defined by the ratio of optical output power to the input power at the SOI waveguide ports, in decibel. The input power is delivered by a tunable laser with 1 nm step scanning in the range of 1260-1630 nm. A manual fiber polarization controller ensures the TE polarization. The grating coupler creates an envelope in the transmittance spectrum making difficult the observation of the resonance. To overcome this issue, the transmittance of the waveguides with a nanoparticles chain was normalized against that of a reference waveguide without plasmonic structures.

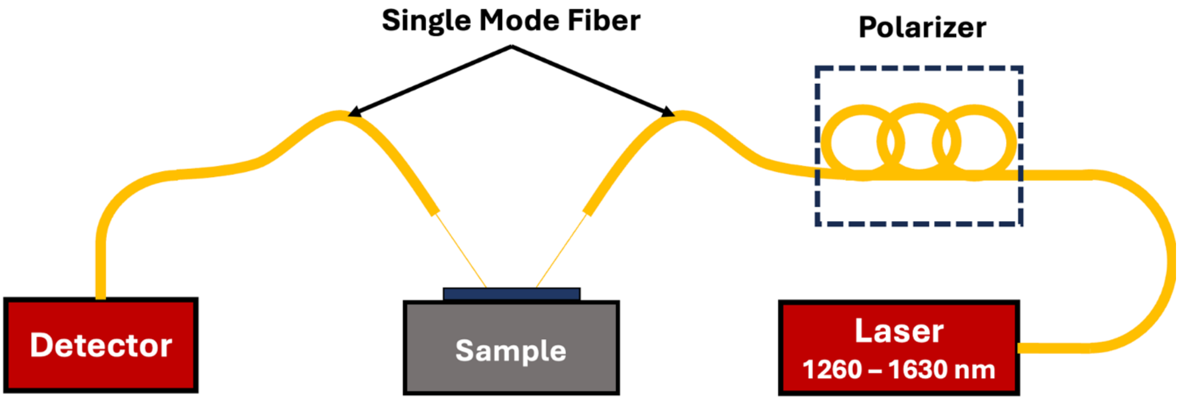

Figure S3.1 : Optical set-up used to perform the measurements. The light from a tunable LASER is polarized to ensure that the TE mode is injected in the waveguide. The fiber and the waveguide are coupled by a grating.

In each sample, straight waveguides are gathered in groups of 6 (Fig. S3.2). One of these waveguides, does not contain gold nanoparticles chain so that its transmittance spectrum exhibits only the grating envelope. The other waveguides have a transmittance spectrum exhibiting the grating envelope and the effect of the chain resonance. Their transmittance spectrum normalizing by the transmittance spectrum of the waveguide without nanoparticles allows the suppression of the grating envelope, and thus makes the chain resonance observable.

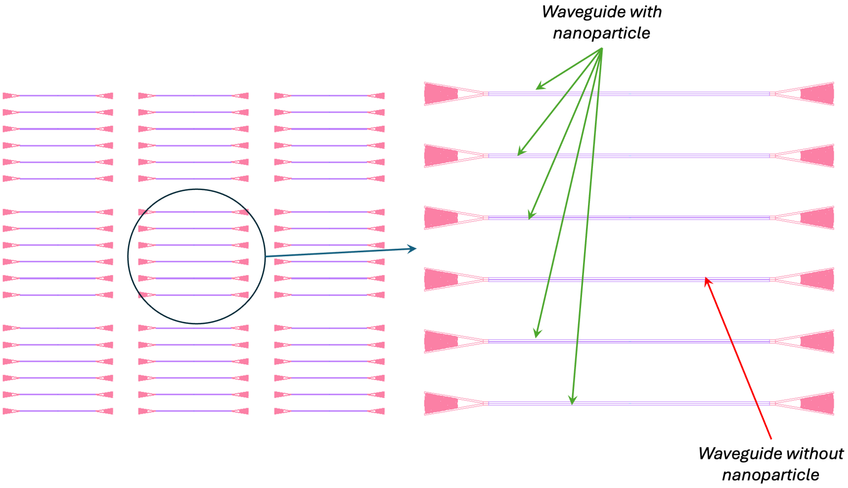

Figure S3.2:Images of the gds file of one sample. The waveguides are gathered by groups of 6 among which only one has no nanoparticle chain. For each group, the transmittance of the waveguides with nanoparticle are normalized by the transmittance of the waveguide without nanoparticle.

In addition, during the encapsulation step of the fabrication process, the cladding height can vary over the sample surface. This may have impact on the grating performance by coupling less light or by changing the diffraction order wavelength. For this reason, each group of 6 waveguides includes a waveguide without nanoparticle, so that each waveguide is normalized by a waveguide with the same grating coupling.

S4: Resonance wavelength dependance on nanoparticle height

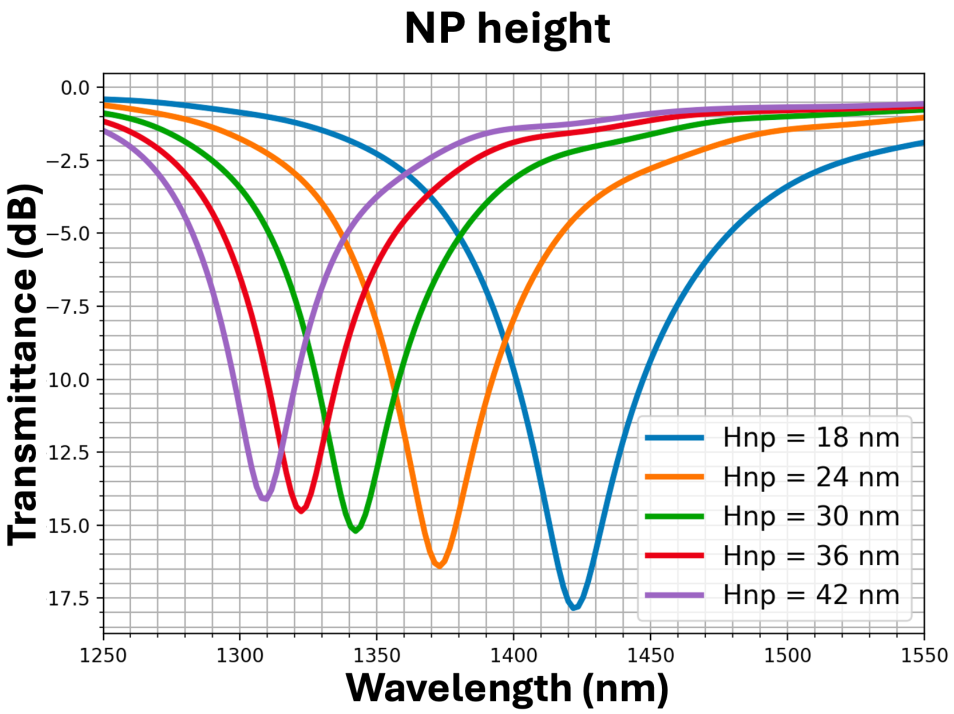

Figure S4: Transmittance spectra for different values of nanoparticle height. The structure considered is a 10-nanoparticle chain with a semi-major axis of 145 nm. The position of the chain is set at hox = 80 nm and y-pos = 0 nm. In the study described in the manuscript, the height of the nanoparticle is set at 30 nm corresponding to a resonance wavelength of 1342 nm. A 6nm decrease of the nanoparticle height leads to a resonance wavelength shift of approximately 30 nm.

S5: Waveguide's reflectance

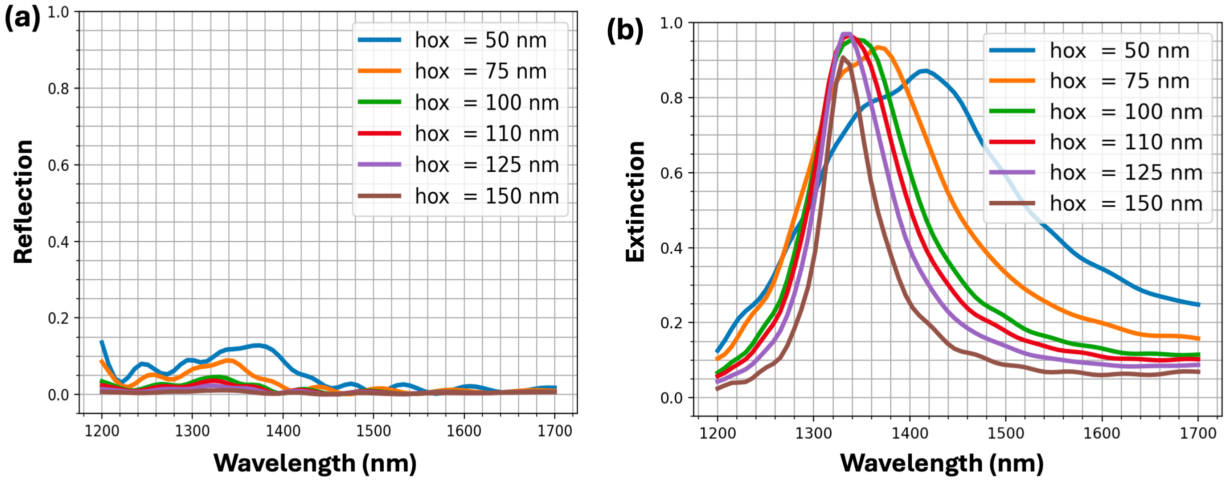

Figure S5:Reflection (a) and extinction (b) spectra of the structure described in Figure 3.5 with a 15*-nanoparticle chain for different values of hox.*

In Figure S4(a), the reflected power for various values of *hox* is depicted. The reflected power decreases as the chain is moved away from the waveguide. The extinction $E = 1 - R - T$ of the system has been calculated where R and T are the reflected and transmitted power (Fig. S4(b)). The extinction exhibits a maximum for the same height where transmittance is at its minimum. Assuming that the diffused power is negligible compared to the power absorbed by the internal loss of the gold, the extinction is equivalent to the absorption. Consequently, the transmittance dip observed at the resonance is only due to the absorption and not due to the reflection or the diffusion of the signal.

S6: Derivation of the Temporal Coupled Mode Theory (TCMT)

Despite the lack of rigorous foundations derived from Maxwell equations[3], TCMT can correctly describe resonant phenomena under certain conditions. These conditions have been derived in the reference[3] where the authors used the quasi-normal modes framework to obtain a rigorous expression of the temporal evolution of the resonator amplitude. This approach shed light on the different assumptions required to apply the TCMT: a slowly varying envelop approximation for the incident light and a local excitation. To respect the former, one needs to have a small overlap between the resonator mode and excitation mode. In our case, the resonator corresponds to the nanoparticle chain supporting the plasmonic mode and TE mode is the incident signal exciting the resonator. Consequently, the interaction between the resonator and the exciting field can occur throughout the entire chain, meaning the local excitation approximation is not well satisfied. For this reason, we are not using the TCMT to find the precise value of the different parameters but as a heuristic tool to gain a deeper understanding of the chain's behavior and to provide an explanation for the variation in transmittance. The energy inside the chain is represented by the amplitude a can be described by the following equations[4,5]:

$$\frac{da}{dt} = (j\omega_0 - \gamma)a + \boldsymbol{\kappa}^{\boldsymbol{T}}\boldsymbol{s}_+ \tag{1.1}$$

$$\boldsymbol{s}_- = \boldsymbol{C}\boldsymbol{s}_+ + \boldsymbol{d}a \tag{1.2}$$

$\boldsymbol{\kappa} = [\kappa_i, \kappa_e]^{\boldsymbol{T}}$ and $\boldsymbol{d} = [d_i, d_e]^{\boldsymbol{T}}$ are vectors that represent the coupling coefficients, as schematized in Figure 5a., $\boldsymbol{s}_{\pm}^{\boldsymbol{T}} = [s_{i\pm}, s_{e\pm}]$ represents the incoming and outgoing channels. The resonator loss is described through the decay rate $\gamma = \gamma_i + \gamma_e$ which is the sum of the intrinsic decay γ_i and the extrinsic decay γ_e. The matrix $\boldsymbol{C}$ describes the scattering matrix of the system without the presence of the chain. We underline the fact that we consider our system reflectionless, this hypothesis is supported by the FDTD simulation where reflection tends to be 0 at resonance (see S4).

Then, we have:

$$\boldsymbol{s}_- = \left(\boldsymbol{C} + \frac{\boldsymbol{\kappa}^{\boldsymbol{T}}\boldsymbol{d}}{j(\omega - \omega_0) + \gamma}\right)\boldsymbol{s}_+ \tag{2}$$

In our model, the resonator is a chain of gold nanoparticles in which the energy flows through the propagating excitation of local surface plasmon resonance in each NP. The intrinsic loss γ_i represents the non-radiative decay of the plasmon into a free electron mode, and/or phonon excitations[6,7]. The extrinsic loss γ_e corresponds to the plasmon decay into photons coupled to the silicon waveguide (radiation outside the system is neglected compared to radiation inside the waveguide).

Because we represent the energy absorption as a channel we can consider that our system is energy conservative and has time reversal symmetry. These two conditions lead to the following equations[5] giving a relationship between the different coupling coefficient vectors:

$$\boldsymbol{C}\boldsymbol{\kappa}^* + \boldsymbol{d} = 0 \tag{3.1}$$

$$\boldsymbol{C}\boldsymbol{d}^{*} + \mathbf{d} = 0 \quad (3.2)$$

$$\boldsymbol{\kappa}^{\dagger}\boldsymbol{\kappa} = \boldsymbol{d}^{\dagger}\boldsymbol{d} = 2\gamma \quad (3.3)$$

To obtain a relation between the different elements of the vector describing the coupling coefficient we considered two ideal cases. The first one, where the system consists of a chain without a waveguide, and the chain already contains energy only decaying through nonradiative processes. By applying the conservation of energy:

$$\frac{d|a|^2}{dt} = -2\gamma_i|a|^2 = -|s_{i-}|^2 = -|d_i a|^2 \quad (4)$$

$$|d_i| = \sqrt{2\gamma_i} \quad (5)$$

By now considering the case where the resonator has no intrinsic loss but only radiative loss, we find that:

$$|d_e| = \sqrt{2\gamma_e} \quad (6)$$

The norms of the coupling vector elements are related to the radiative and non-radiative decay of the resonators. To get a complete description of these elements, one needs to derive an expression of the matrix C. This matrix describes the background scattering matrix. So, in the absence of resonator the output and input channels are related through:

$$\boldsymbol{s}_{-} = \boldsymbol{C}\boldsymbol{s}_{+} \quad (7)$$

We assume that the two channels representing the different decaying mechanisms of the resonator are independent leading to $C_{12} = C_{21} = 0$. By considering the loss of the waveguide lossless, $s_{e-} = s_{e+}$, then

$$\boldsymbol{C} = \begin{pmatrix} 1 & 0 \\ 0 & 1 \end{pmatrix} \quad (8)$$

By injecting $\boldsymbol{C}$ in the equations (3) one finds:

$$d_e = \kappa_e = j\sqrt{2\gamma_e} \quad (9.1)$$

$$d_i = \kappa_i = j\sqrt{2\gamma_i} \quad (9.2)$$

Having defined all the parameters we can express the equation (2) by considering that in our system we have $s_{i+} = 0$, assuming a harmonic dependence of the excitation ($s_{+} = s_{+0}e^{j\omega t}$) and searching the harmonic solution ($a = a_0 e^{j\omega t}$)

$$\begin{pmatrix} s_{i-} \\ s_{e-} \end{pmatrix} = \left[\begin{pmatrix} 1 & 0 \\ 0 & 1 \end{pmatrix} + \frac{1}{j(\omega - \omega_0) + \gamma}\begin{pmatrix} d_i\kappa_i & d_i\kappa_e \\ \kappa_i d_e & d_e\kappa_e \end{pmatrix}\right]\begin{pmatrix} 0 \\ s_{e+} \end{pmatrix} \quad (10)$$

We find

$$\begin{pmatrix} s_{i-} \\ s_{e-} \end{pmatrix} = \begin{pmatrix} -\frac{2\sqrt{\gamma_e \gamma_i}}{j(\omega - \omega_0) + \gamma} s_{e+} \\ \left(1 - \frac{2\gamma_e}{j(\omega - \omega_0) + \gamma}\right) s_{e+} \end{pmatrix} \tag{11}$$

Then, we can derive an expression for the chain's absorbance A and the waveguide's transmittance T:

$$A = \left|\frac{s_{i-}}{s_{e+}}\right|^2 = \left|-\frac{2\sqrt{\gamma_e \gamma_i}}{j(\omega - \omega_0) + \gamma}\right|^2 \tag{12.1}$$

$$T = \left|\frac{s_{e-}}{s_{e+}}\right|^2 = \left|1 - \frac{2\gamma_e}{j(\omega - \omega_0) + \gamma}\right|^2 \tag{12.2}$$

At resonance ($\omega = \omega_0$), it becomes:

$$A = \left|\frac{2\sqrt{\gamma_e \gamma_i}}{\gamma_\mathrm{i} + \gamma_e}\right|^2 \tag{13.1}$$

$$T = \left|\frac{\gamma_\mathrm{i} - \gamma_e}{\gamma_\mathrm{i} + \gamma_e}\right|^2 \tag{13.2}$$

When the external and internal decays are equal ($\gamma_e = \gamma_i$), the system is in the critical coupling configuration: the absorbance reaches its maximum ($A = 1$) and the transmittance is $T = 0$.

The phase of the transmittance can be expressed as:

$$\phi = Arctan\left(\frac{2\gamma_e(\omega - \omega_0)}{\gamma_i^2 - \gamma_e^2 + (\omega - \omega_0)^2}\right) \tag{14}$$

REFERENCES

(1) Liu, Q.; Zhou, P. Effect of Recessed Chuck Slits on the Edge Buildup in Spin Coating for Rectangular Substrates. *Progress in Organic Coatings* **2025**, *200*, 108994. https://doi.org/10.1016/j.porgcoat.2024.108994.

(2) Arscott, S. The Limits of Edge Bead Planarization and Surface Levelling in Spin-Coated Liquid Films. *J. Micromech. Microeng.* **2020**, *30* (2), 025003. https://doi.org/10.1088/1361-6439/ab60be.

(3) Wu, T.; Lalanne, P. Exact Maxwell Evolution Equation of Resonator Dynamics: Temporal Coupled-Mode Theory Revisited. *Opt. Express* **2024**, *32* (12), 20904. https://doi.org/10.1364/OE.517237.

(4) Fan, S.; Suh, W.; Joannopoulos, J. D. Temporal Coupled-Mode Theory for the Fano Resonance in Optical Resonators. *J. Opt. Soc. Am. A, JOSAA* **2003**, *20* (3), 569–572. https://doi.org/10.1364/JOSAA.20.000569.

(5) Zhao, Z.; Guo, C.; Fan, S. Connection of Temporal Coupled-Mode-Theory Formalisms for a Resonant Optical System and Its Time-Reversal Conjugate. *Phys. Rev. A* **2019**, *99* (3), 033839. https://doi.org/10.1103/PhysRevA.99.033839.

(6) Heilweil, E. J.; Hochstrasser, R. M. Nonlinear Spectroscopy and Picosecond Transient Grating Study of Colloidal Gold. *The Journal of Chemical Physics* **1985**, *82* (11), 4762–4770. https://doi.org/10.1063/1.448693.

(7) Sönnichsen, C.; Franzl, T.; Wilk, T.; Von Plessen, G.; Feldmann, J.; Wilson, O.; Mulvaney, P. Drastic Reduction of Plasmon Damping in Gold Nanorods. *Phys. Rev. Lett.* **2002**, *88* (7), 077402. https://doi.org/10.1103/PhysRevLett.88.077402.
